# Determination of essential biomarkers in lung cancer: a real-world data study in Spain with demographic, clinical, epidemiological and pathological characteristics

**DOI:** 10.1186/s12885-022-09830-8

**Published:** 2022-07-05

**Authors:** Mariano Provencio, Manuel Cobo, Delvys Rodriguez-Abreu, Virginia Calvo, Enric Carcereny, Alexandra Cantero, Reyes Bernabé, Gretel Benitez, Rafael López Castro, Bartomeu Massutí, Edel del Barco, Rosario García Campelo, Maria Guirado, Carlos Camps, Ana Laura Ortega, Jose Luis González Larriba, Alfredo Sánchez, Joaquín Casal, M. Angeles Sala, Oscar Juan-Vidal, Joaquim Bosch-Barrera, Juana Oramas, Manuel Dómine, Jose Manuel Trigo, Remei Blanco, Julia Calzas, Idoia Morilla, Airam Padilla, Joao Pimentao, Pedro A. Sousa, Maria Torrente

**Affiliations:** 1 Medical Oncology Department, Hospital Universitario Puerta de Hierro-Majadahonda, C/ Manuel de Falla 1, Majadahonda, 28222 Madrid, Spain; 2 Hospital Regional Universitario de Málaga, Málaga, Spain; 3 Hospital Universitario Insular de Gran Canaria, Las Palmas de Gran Canaria, Spain; 4 Catalan Institute of Oncology, Hospital Universitari Germans Trias i Pujol, B-ARGO, IGTP, Badalona, Spain; 5 Hospital Universitario Virgen del Rocio, Sevilla, Spain; 6 Hospital Clínico Universitario de Valladolid, Valladolid, Spain; 7 Hospital General Universitario de Alicante, Alicante, Spain; 8 Hospital Universitario de Salamanca, Salamanca, Spain; 9 Complejo Hospitalario Universitario A Coruña, A Coruña, Spain; 10 Hospital General Universitario de Elche, Elche, Spain; 11 Hospital General Universitario de Valencia, Valencia, Spain; 12 Universidad de Valencia, Valencia, Spain; 13 CIBERONC, Madrid, Spain; 14 Hospital Universitario de Jaén, Jaén, Spain; 15 Hospital Universitario Clínico San Carlos, Madrid, Spain; 16 Hospital Provincial de Castellón, Castellón, Spain; 17 Complexo Hospitalario de Vigo, Vigo, Spain; 18 OSI Bilbao Basurto, Bibao, Spain; 19 Hospital Universitario y Politécnico La Fe, Valencia, Spain; 20 Catalan Institute of Oncology, Hospital Universitari Dr. Josep Trueta, Girona, Spain; 21 Hospital Universitario de Canarias, Santa Cruz de Tenerife, Spain; 22 Hospital Universitario Fundación Jiménez Díaz. IIS-FJD, Madrid, Spain; 23 Hospital Universitario Virgen de la Victoria, Málaga, Spain; 24 Consorci Sanitari de Terrassa, Barcelona, Spain; 25 Hospital Universitario de Fuenlabrada, Fuenlabrada, Spain; 26 Complejo Hospitalario de Navarra, Navarra, Spain; 27 Hospital Universitario Nuestra Señora de la Candelaria, Santa Cruz de Tenerife, Spain; 28 Department of Electrical Engineering, NOVA School of Science and Technology, Universidade Nova de Lisboa, Lisbon, Portugal

**Keywords:** Biomarkers, Testing, Metastatic lung cancer, Targeted therapies

## Abstract

**Background:**

The survival of patients with lung cancer has substantially increased in the last decade by about 15%. This increase is, basically, due to targeted therapies available for advanced stages and the emergence of immunotherapy itself. This work aims to study the situation of biomarker testing in Spain.

**Patients and methods:**

The Thoracic Tumours Registry (TTR) is an observational, prospective, registry-based study that included patients diagnosed with lung cancer and other thoracic tumours, from September 2016 to 2020. This TTR study was sponsored by the Spanish Lung Cancer Group (GECP) Foundation, an independent, scientific, multidisciplinary oncology society that coordinates more than 550 experts and 182 hospitals across the Spanish territory.

**Results:**

Nine thousand two hundred thirty-nine patients diagnosed with stage IV non-small cell lung cancer (NSCLC) between 2106 and 2020 were analysed. 7,467 (80.8%) were non-squamous and 1,772 (19.2%) were squamous. Tumour marker testing was performed in 85.0% of patients with non-squamous tumours vs 56.3% in those with squamous tumours (*p*-value < 0.001). The global testing of EGFR, ALK, and ROS1 was 78.9, 64.7, 35.6% respectively, in non-squamous histology. PDL1 was determined globally in the same period (46.9%), although if we focus on the last 3 years it exceeds 85%. There has been a significant increase in the last few years of all determinations and there are even close to 10% of molecular determinations that do not yet have targeted drug approval but will have it in the near future. 4,115 cases had a positive result (44.5%) for either EGFR, ALK, KRAS, BRAF, ROS1, or high PDL1.

**Conclusions:**

Despite the lack of a national project and standard protocol in Spain that regulates the determination of biomarkers, the situation is similar to other European countries. Given the growing number of different determinations and their high positivity, national strategies are urgently needed to implement next-generation sequencing (NGS) in an integrated and cost-effective way in lung cancer.

## Background

Lung cancer has evolved from being considered a single disease with few treatment options, to represent a complex disease with many effective treatments. The management and treatment choice depend on the tumour molecular profile.

The survival of patients with lung cancer has substantially increased in the last decade by about 15%. This increase is, basically, due to targeted therapies available for advanced stages and the emergence of immunotherapy itself [1].

The Thoracic Tumours Registry (TTR) was created in 2016 and since then data from more than 180 hospitals throughout Spain has been collected. Several epidemiological and clinical aspects of it have already been studied [2–4]. New biomarkers linked to the use of targeted therapies are progressively being incorporated in the clinical practice while, at the same time, there is a growing concern within oncologists that all patients may have access to both essential accurate diagnoses and targeted treatments without delay after regulatory approval. However, our perception is that this process does not develop homogeneously and synchronously. In Spain, despite guidelines recommendations, it is unknown to date what percentage of patients undergo biomarker determination and will therefore have access to the most appropriate treatment. More importantly, there is a lack of political consensus from the health authorities on precision medicine. The request of these tests relies on each physician, based on existing evidence and their own experience. Therefore, a great field of uncertainty in this aspect needs to be addressed and explored.

The main objective of this study was to analyse the different characteristics between squamous vs. non-squamous histology in metastatic non-small cell lung cancer (NSCLC), and the proportion of patients who had a molecular determination and the positivity rate.

## Methods

### Study design and population

The TTR is an observational, prospective, registry-based study that enrolled patients diagnosed with lung cancer and other thoracic tumours from September 2016 to date. The study was conducted in accordance with the Declaration of Helsinki. The registry was classified by the Spanish Agency for Drugs and Medical Devices (AEMPS) in 2016, and it is registered on the ClinicalTrials.gov database (NCT02941458). Protocol approval was obtained from the institutional ethics committee at Puerta de Hierro-Majadahonda University Hospital (No. PI 148/15).

This TTR study was sponsored by the GECP, an independent, multidisciplinary oncology group that coordinates more than 550 experts and 182 hospitals across the Spanish territory. The registry creation was proposed by the steering committee with the aim to promote lung cancer research and incorporate treatment advances into clinical practice.

For this analysis, patients with histologically confirmed stage IV NSCLC were included regardless of sex, age, and type of treatment (active treatment or palliative). All patients provided signed informed consent before inclusion in the TTR.

### Variables and outcomes

Research teams collected data from patient electronic health records using an electronic data capture system (EDC). Sociodemographic, epidemiological, clinical, molecular and treatment outcome variables were recorded in an Electronic Case Report form (eCRF). The information was classified into the following categories: (I) patient personal history, which included sex, age at diagnosis, performance status (PS), tobacco consumption, and comorbidities; (II) diagnosis, including histological subtype, TNM classification of the tumour and location of metastases; (III) molecular profiling of the tumour; (IV) treatment patterns (surgery, chemotherapy, radiotherapy); (V) response and survival, including response rates, overall survival (OS) and progression-free survival (PFS); and (VI) prognostic factors.

### Statistical analysis

Descriptive statistics were performed and quantitative data were summarized as mean, standard deviation (SD) interquartile range, minimum and maximum. Qualitative variables were summarized as frequencies and percentages in the entire cohort. Characteristics of the two groups (squamous and non-squamous) were compared using the chi-squared test for categorical variables. The significance level was established at a value of 0.05.

## Results

We analysed 9,239 patients included in TTR with stage IV NSCLC. Of these, 7,467 (80.8%) were no squamous and 1,772 (19.2%) were squamous. Of the 7,467 with a non-squamous tumour, 6,585 (88%) were adenocarcinoma and the rest other varieties or NOS.

Table 1 provides a description of the demographic characteristics of the patients, both for the total cohort and for each of the two histological groups. Patients with squamous tumours present a significantly higher percentage of males (86.8% vs 68.1%, *p*-value < 0.001), a higher mean age (67.5 vs 63.6, *p*-value < 0.001), a lower presence of non-smokers (3.8% vs 17.3%, *p*-value < 0.001) and a lower percentage of ECOG 0 (23.1% vs 26.5%, *p*-value = 0.013). It also shows the distribution by autonomous community.

**Table 1:** Characteristics of the patients

	Total	Non squamous	Squamous
Sex			
Men	6.623 (71,7%)	5.085 (68,1%)	1.538 (86,8%)
Women	2.616 (28,3%)	2.382 (31,9%)	234 (13,2%)
Age at diagnosis			
Mean (DT)	64,3 (10,5)	63,6 (10,6)	67,5 (9,0)
Median (RIQ)	65 (57–72)	64 (56–71)	68 (62–74)
< 55 years old	1.538 (17,4%)	1.405 (19,6%)	133 (7,9%)
55–64 years old	2.821 (31,9%)	2.341 (32,7%)	480 (28,5%)
65–74 years old	2.961 (33,5%)	2.262 (31,6%)	699 (41,4%)
> = 75 years old	1.520 (17,2%)	1.145 (16,0%)	375 (22,2%)
Smoking habit			
Never smoker	1.344 (14,5%)	1.277 (17,3%)	67 (3,8%)
Former smoker	3.989 (43,2%)	3.133 (41,6%)	856 (49,0%)
Current smoker	3.776 (40,9%)	2.953 (40,1%)	823 (47,1%)
Performance Status			
ECOG 0	2.385 (25,8%)	1.976 (26,5%)	409 (23,1%)
ECOG 1	4.976 (53,9%)	3.989 (53,5%)	987 (55,7%)
ECOG > = 2	1.869 (20,2%)	1.494 (20,0%)	375 (21,2%)
Autonomous community			
Andalucía	1.930 (20,9%)	1.448 (19,4%)	482 (27,2%)
Balears	26 (0,3%)	23 (0,3%)	3 (0,2%)
Canarias	1.217 (13,2%)	1.013 (13,6%)	204 (11,5%)
Castilla y León	815 (8,8%)	667 (8,9%)	148 (8,4%)
Castilla-La Mancha	170 (1,8%)	116 (1,6%)	54 (3,0%)
Cataluña	1.035 (11,2%)	848 (11,4%)	187 (10,6%)
Comunidad Valenciana	1.379 (14,9%)	1.143 (15,3%)	236 (13,3%)
Extremadura	72 (0,8%)	52 (0,7%)	20 (1,1%)
Galicia	563 (6,1%)	475 (6,4%)	88 (5,0%)
Madrid	1.616 (17,5%)	1.336 (17,9%)	280 (15,8%)
Murcia	80 (0,9%)	65 (0,9%)	15 (0,8%)
Navarra	130 (1,4%)	110 (1,5%)	20 (1,1%)
País Vasco	206 (2,2%)	171 (2,3%)	35 (2,0%)

Table 2 shows the presence of comorbidities for patients in whom this characteristic has been recorded. Patients with squamous tumours present a significantly higher presence of comorbidities than non-squamous (88.3% vs 81.3%). When analysing the different comorbidities, we observe a significantly higher presence of heart disease (19.9% vs 13.5%), diabetes mellitus (24.4% vs 17.4%), chronic obstructive pulmonary disease (COPD) (29.6% vs 15.3%), former alcoholism (8.5% vs 6.9%), hypertension (47.7% vs 40.8%), and vascular disease (6.9% vs 5.1%) in the group of patients with squamous tumours compared to non-squamous ones.

**Table 2:** Baseline comorbidities of the patients

	Total	Non-squamous	Squamous	*p*-value
Comorbidities				< 0,001
None	1.530 (17,4%)	1.334 (18,7%)	196 (11,7%)	
At least one	7.266 (82,6%)	5.785 (81,3%)	1.481 (88,3%)	
Hypertension (HT)	3.704 (42,1%)	2.904 (40,8%)	800 (47,7%)	< 0,001
Dyslipidemia	2.499 (28,4%)	1.992 (28,0%)	507 (30,2%)	0,066
Diabetes Mellitus (DM)	1.652 (18,8%)	1.242 (17,4%)	410 (24,4%)	< 0,001
Chronic Obstructive Pulmonary Disease (COPD)	1.588 (18,1%)	1.092 (15,3%)	496 (29,6%)	< 0,001
Cardiomyopathy	1.293 (14,7%)	960 (13,5%)	333 (19,9%)	< 0,001
Depression/Anxiety	642 (7,3%)	543 (7,6%)	99 (5,9%)	0,014
Former Alcoholism	635 (7,2%)	493 (6,9%)	142 (8,5%)	0,031
Hypercholesterolemia	619 (7,0%)	498 (7,0%)	121 (7,2%)	0,750
Vasculopathy	481 (5,5%)	366 (5,1%)	115 (6,9%)	0,007
Obesity	333 (3,8%)	259 (3,6%)	74 (4,4%)	0,136
Nephropathy	236 (2,7%)	179 (2,5%)	57 (3,4%)	0,053
Hepatitis	177 (2,0%)	140 (2,0%)	37 (2,2%)	0,500
Asthma	171 (1,9%)	145 (2,0%)	26 (1,6%)	0,237
Tuberculosis	137 (1,6%)	110 (1,5%)	27 (1,6%)	0,827
Other	3.881 (44,1%)	3065 (43,1%)	816 (48,7%)	< 0,001

The different characteristics related to the tumour are described in Table 3. No differences were detected in the percentage of patients showing metastasis at diagnosis (*p*-value = 0.333). There is a greater number of metastatic locations in the group of patients with non-squamous tumours (*p*-value < 0.001). If we analyse the possible differences in the locations for the group of patients with metastases, significantly higher percentages are observed in the group of patients with non-squamous tumours. These were patients that presented metastases in bone (38.8% vs 31.8%, *p*-value < 0.001), adrenal (19.2% vs 16.1%, *p*-value = 0.004), central nervous system (22.1% vs 10.1%, *p*-value < 0.001), pleural effusion (19.8% vs 15.1%, *p*-value < 0.001), and pericardial effusion (3.6% vs 2.5%, *p*-value = 0.023), while there is a significantly lower percentage in lung metastases (39.8% vs 43, 6%, *p*-value = 0.006).

**Table 3:** Characteristics of the tumours

	Total	Non-Squamous	Squamous	*p*-value
Metastasis upon diagnosis				0,333
No	111 (1,3%)	86 (1,2%)	25 (1,5%)	
Yes	8604 (98,7%)	6968 (98,8%)	1636 (98,5%)	
Number of metastatic sites				
Mean (DT)	2,21 (1,31)	2,26 (1,34)	2,00 (1,20)	< 0,001
1	3.189 (37,1%)	2.490 (35,7%)	699 (42,7%)	
2	2.547 (29,6%)	2.020 (29,0%)	527 (32,2%)	
3	1.551 (18,0%)	1.311 (18,8%)	240 (14,7%)	
4	796 (9,3%)	696 (10,0%)	100 (6,1%)	
> =5	521 (6,1%)	451 (6,5%)	70 (4,3%)	
Liver	1.385 (16,1%)	1.120 (16,1%)	265 (16,2%)	> 0,9
Bone	3.224 (37,5%)	2.704 (38,8%)	520 (31,8%)	< 0,001
Thoracic lymphadenopathy	2.063 (24,0%)	1.686 (24,2%)	377 (23,0%)	0,335
Lung	3.487 (40,5%)	2.774 (39,8%)	713 (43,6%)	0,006
Non thoracic lymphadenopathy	1.203 (14,0%)	999 (14,3%)	204 (12,5%)	0,052
Adrenal	1.600 (18,6%)	1.336 (19,2%)	264 (16,1%)	0,004
Central Nervous System	1.707 (19,8%)	1.542 (22,1%)	165 (10,1%)	< 0,001
Pleural Effusion	1.628 (18,9%)	1.381 (19,8%)	247 (15,1%)	< 0,001
Pleural nodules	802 (9,3%)	657 (9,4%)	145 (8,9%)	0,508
Peritoneal cavity	233 (2,7%)	189 (2,7%)	44 (2,7%)	> 0,9
Pericardial effusion	294 (3,4%)	253 (3,6%)	41 (2,5%)	0,023
Pancreas	98 (1,1%)	80 (1,1%)	18 (1,1%)	> 0,9
Bilateral Lymphangitis	198 (2,3%)	170 (2,4%)	28 (1,7%)	0,082
Soft tissues	347 (4,0%)	277 (4,0%)	70 (4,3%)	0,576
Subcutaneous	130 (1,5%)	103 (1,5%)	27 (1,7%)	0,575
Meningeal carcinomatosis	26 (0,3%)	24 (0,3%)	2 (0,1%)	0,208
Stage T				0,056
T1	797 (8,6%)	712 (9,5%)	85 (4,8%)	< 0,001
T1a	201 (2,2%)	179 (2,4%)	22 (1,2%)	
T1b	385 (4,2%)	343 (4,6%)	42 (2,4%)	
T1c	211 (2,3%)	190 (2,5%)	21 (1,2%)	
T2	1666 (18,0%)	1.388 (18,6%)	278 (15,7%)	
T2a	1105 (12,0%)	922 (12,3%)	183 (10,3%)	
T2b	561 (6,1%)	466 (6,2%)	95 (5,4%)	
T3	1.458 (15,8%)	1.162 (15,6%)	296 (16,7%)	
T4	3.056 (33,1%)	2.294 (30,7%)	762 (43%)	
Stage N				
NX	2.369 (25,6%)	1.968 (26,4%)	401 (22,6%)	
N0	1.011 (10,9%)	820 (11%)	191 (10,8%)	
N1	660 (7,1%)	530 (7,1%)	130 (7,3%)	< 0,001
N2	2.556 (27,7%)	1.992 (26,7%)	564 (31,8%)	
N3	2.643 (28,6%)	2.157 (28,9%)	486 (27,4%)	
Stage M				
M1a	2.568 (27,8%)	1.978 (26,5%)	590 (33,3%)	
M1b	3.626 (39,2%)	2.940 (39,4%)	686 (38,7%)	
M1c	2.270 (24,6%)	1.925 (25,8%)	345 (19,5%)	< 0,001

In Table 4 we report the types of tumour markers analysed in both groups of patients. Any of the available tumour markers was performed in 85.0% of non-squamous tumours vs 56.3% in squamous tumours (*p*-value < 0.001). Specifically, EGFR was analysed in 78.9% of patients with non-squamous tumour versus 16.7% of patients with squamous tumour (*p*-value < 0.001). Additionally, a higher percentage of EGFR positive tests was observed in patients with non-squamous tumours (18.5% vs 7.9%, *p*-value < 0.001).

**Table 4:** Tumour biomarkers tested and obtained results

	Total	Non-Squamous	Squamous	*p*-value
Tumour biomarker				
None	1.896 (20,5%)	1.122 (15,0%)	774 (43,7%)	
Any	7.343 (79,5%)	6.345 (85,0%)	998 (56,3%)	< 0,001
EGFR				
Not tested	3.050 (33,0%)	1.574 (21,1%)	1.476 (83,3%)	
Tested	6.189 (67,0%)	5.893 (78,9%)	296 (16,7%)	< 0,001
Unknown	26 (0,4%)	22 (0,4%)	4 (1,4%)	
Negative	5.053 (81,6%)	4.784 (81,2%)	269 (90,9%)	
Positive	1.110 (17,9%)	1.087 (18,4%)	23 (7,8%)	< 0,001
T790M +	32 (2,9%)	30 (2,8%)	2 (8,7%)	
T790M -	72 (6,5%)	72 (6,6%)	0 (0,0%)	
Exon 19	594 (53,5%)	580 (53,4%)	14 (60,9%)	
Exon 20	69 (6,2%)	65 (6,0%)	4 (17,4%)	
Exon 21	346 (31,2%)	342 (31,5%)	4 (17,4%)	
NOS	35 (3,2%)	35 (3,2%)	0 (0,0%)	
Other	87 (7,8%)	85 (7,8%)	2 (8,7%)	
ALK				
Not tested	4.166 (45,1%)	2.638 (35,3%)	1.528 (86,2%)	
Tested	5.073 (54,9%)	4.829 (64,7%)	244 (13,8%)	< 0,001
Negative	4.817 (95,0%)	4.576 (94,8%)	241 (98,8%)	
Positive	256 (5,0%)	253 (5,2%)	3 (1,2%)	0,002
KRAS				
Not tested	8.535 (92,4%)	6.809 (91,2%)	1.726 (97,4%)	
Tested	704 (7,6%)	658 (8,8%)	46 (2,6%)	< 0,001
Undetectable	484 (68,8%)	447 (67,9%)	37 (80,4%)	
Detectable	220 (31,3%)	211 (32,1%)	9 (19,6%)	0,099
BRAF				
Not tested	8.116 (87,8%)	6.411 (85,9%)	1.705 (96,2%)	
Tested	1.123 (12,2%)	1.056 (14,1%)	67 (3,8%)	< 0,001
Undetectable	1.061 (94,5%)	994 (94,1%)	67 (100%)	
Detectable	62 (5,5%)	62 (5,9%)	0 (0%)	0,046
ROS1				
Not tested	6.438 (69,7%)	4.808 (64,4%)	1.630 (92,0%)	
Tested	2.801 (30,3%)	2.659 (35,6%)	142 (8,0%)	< 0,001
Negative	2.718 (97,0%)	2.580 (97,0%)	138 (97,2%)	
Positive	83 (3,0%)	79 (3,0%)	4 (2,8%)	–
FGFR1				
Not tested	9.182 (99,4%)	7.416 (99,3%)	1.766 (99,7%)	
Tested	57 (0,6%)	51 (0,7%)	6 (0,3%)	0,127
Not amplified	54 (94,7%)	49 (96,1%)	5 (83,3%)	
Amplified	3 (5,3%)	2 (3,9%)	1 (16,7%)	–
PDL1				
Not tested	4.903 (53,1%)	3.987 (53,4%)	916 (51,7%)	
Tested	4.336 (46,9%)	3.480 (46,6%)	856 (48,3%)	0,204
Unknown	176 (4,1%)	132 (3,8%)	44 (5,1%)	
Negative	1.812 (41,8%)	1.489 (42,8%)	323 (37,7%)	
Positive	2.348 (54,2%)	1.859 (53,4%)	489 (57,1%)	0,016
Unknown	79 (3,4%)	56 (3,0%)	23 (4,7%)	
< 50%	1.150 (49,0%)	888 (47,8%)	262 (53,6%)	
> =50%	1.119 (47,7%)	915 (49,2%)	204 (41,7%)	
HER2				
Not tested	9.119 (98,7%)	7.357 (98,5%)	1.762 (99,4%)	
Tested	120 (1,3%)	110 (1,5%)	10 (0,6%)	0,001
Undetectable	110 (91,7%)	100 (90,9%)	10 (100%)	
Detectable	10 (8,3%)	10 (9,1%)	0 (0%)	–
RET				
Not tested	9.097 (98,5%)	7.336 (98,2%)	1.761 (99,4%)	
Tested	142 (1,5%)	131 (1,8%)	11 (0,6%)	< 0,001
No translocated	136 (95,8%)	125 (95,4%)	11 (100,0%)	
Translocated	6 (4,2%)	6 (4,6%)	0 (0,0%)	–
MET				
Not tested	8.998 (97,4%)	7.238 (96,9%)	1.760 (99,3%)	
Tested	241 (2,6%)	229 (3,1%)	12 (0,7%)	< 0,001
Negative	218 (90,5%)	206 (90,0%)	12 (100,0%)	
Amplified	18 (7,5%)	18 (7,9%)	0 (0,0%)	
Mutated	3 (1,2%)	3 (1,3%)	0 (0,0%)	0,612
Overexpressed	2 (0,8%)	2 (0,9%)	0 (0,0%)	
NTRK				
Not tested	9.098 (98,5%)	7.336 (98,2%)	1.762 (99,4%)	
Tested	141 (1,5%)	131 (1,8%)	10 (0,6%)	< 0,001
Negative	140 (99,3%)	130 (99,2%)	10 (100,0%)	
Positive	1 (0,7%)	1 (0,8%)	0 (0,0%)	–
Other biomarkers				
Not tested	8.853 (95,8%)	7.144 (95,7%)	1.709 (96,4%)	
Tested	386 (4,2%)	323 (4,3%)	63 (3,6%)	0,165

ALK was analysed in 64.7% of patients with non-squamous tumour compared to 13.8% of patients with squamous tumour (*p*-value < 0.001), with a higher percentage of positive tests being observed in patients with non-squamous tumours (5.2% vs 1.2%, *p*-value = 0.002). ROS1 was performed in 35.6% of non-squamous patients compared to 8.0% of squamous patients (*p*-value < 0.001), although the percentage of positives was similar in both groups.

KRAS was analysed in 8.8% of patients with non-squamous tumour compared to 2.6% of patients with squamous tumour (*p*-value < 0.001), with positive cases in the non-squamous group (32.1% vs 19.6%) that almost reach statistical significance (*p*-value = 0.099).

BRAF was analysed in 14.1% of patients with non-squamous tumour versus only 3.8% of patients with squamous tumour (*p*-value < 0.001), with a higher percentage of positive cases in patients with non-squamous tumours (5.9% vs 0.0%, *p*-value = 0.046) who were tested.

Despite the low number of patients tested for HER2, the percentage of testing was higher in the group of non-squamous patients (1.5% vs 0.6%, *p*-value = 0.001). Similar results were observed in the RET (1.8% in non-squamous vs 0.6% squamous, *p*-value < 0.001), MET (3.1% in non-squamous vs 0.7% squamous, *p*- value < 0.001) or NTRK tests (1.8% in non-squamous vs 0.6% squamous, *p*-value < 0.001).

No differences were observed in the performance of other types of tests. On the other hand, no differences were reported in the rates of PDL1 testing between both groups, although there was a higher percentage of positive cases within the squamous group (60.2% vs 55.5%, *p*-value = 0.016).

**Fig. 1:**
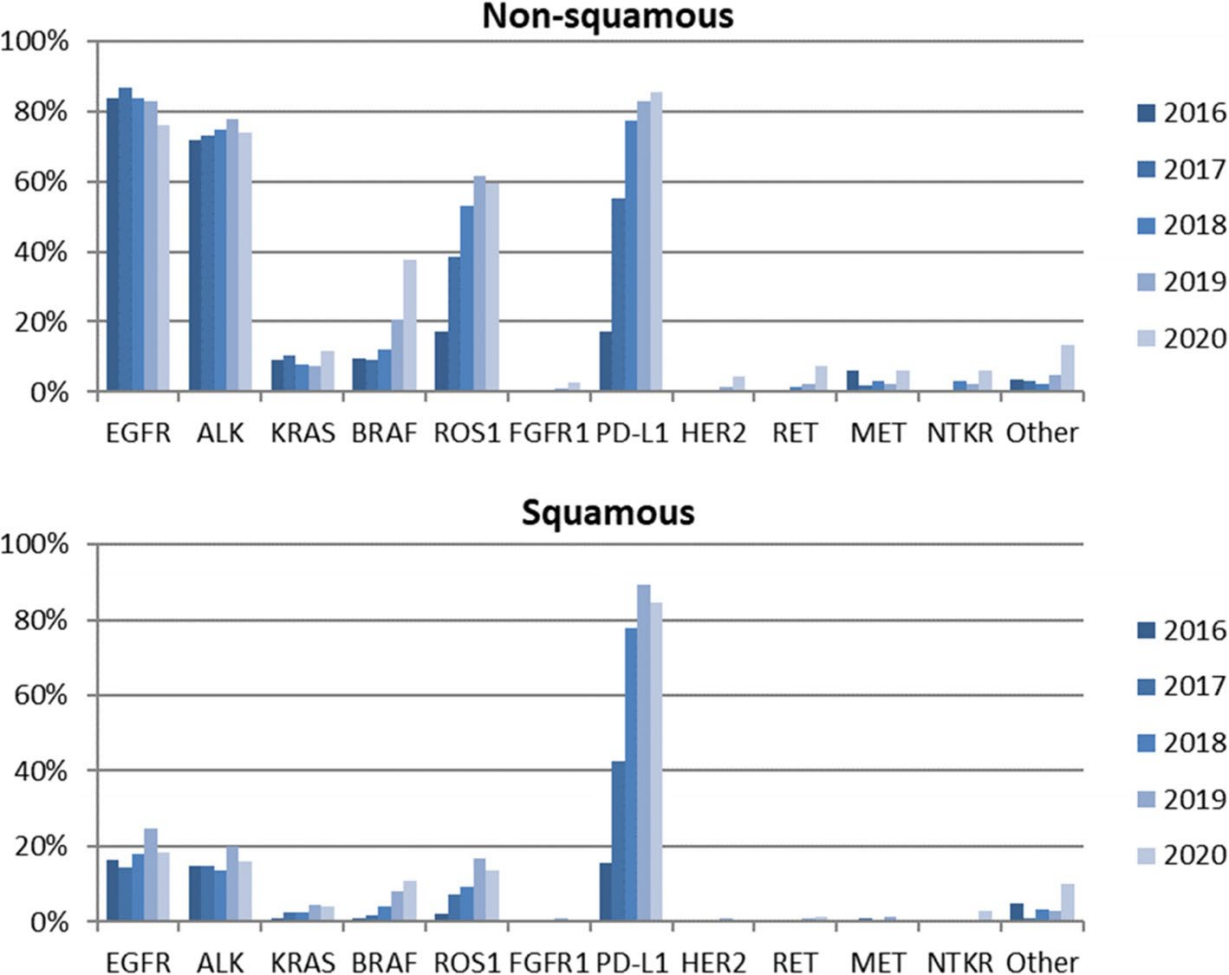
Test rate of tumour markers analysed between 2016 and 2020 according to histological type

In Fig. 1 the percentage of patients who have undergone each test is depicted, according to the year of diagnosis (2016–2020) and the histological type. Important differences between both histological groups were observed for some of the biomarkers tested, such as EGFR, ALK or ROS1.

Moreover, Table 5 shows the percentage of tests performed for the ALK and ROS1 markers in these patients, both globally and according to histological type. A higher rate of tests performed associated with the ALK marker is observed in non-squamous patients (84.6% vs 74.7%, *p*-value < 0.001), in addition to a higher percentage of positive results (5.2% vs 1.0%, *p*-value = 0.004), whereas ROS1 testing concluded similar percentages of tested patients or with positive results.

**Table 5:** Analysis and results of ALK and ROS1 markers in EGFR non-mutated patients

	Total	Non-squamous	Squamous	*p*-value
ALK				
Not analysed	803 (15,9%)	735 (15,4%)	68 (25,3%)	
Analysed	4.250 (84,1%)	4.049 (84,6%)	201 (74,7%)	< 0,001
Negative	4.039 (95,0%)	3.840 (94,8%)	199 (99,0%)	
Positive	211 (5,0%)	209 (5,2%)	2 (1,0%)	0,004
ROS1				
Not analysed	2.702 (53,5%)	2.557 (53,4%)	145 (53,9%)	
Analysed	2.351 (46,5%)	2.227 (46,6%)	124 (46,5%)	0,900
Negative	2.284 (97,2%)	2.164 (97,2%)	120 (96,8%)	
Positive	67 (2,8%)	63 (2,8%)	4 (3,2%)	0,778

## Discussion

In the last few years, there has been a significant increase in the determinations of all molecular biomarkers in lung cancer. Of note, approximately 10% of molecular determinations lack targeted drug approval but will have it soon. The data shows that 85.0% of the patients with non-squamous tumours and 56.3% with squamous tumours were tested for essential biomarkers for treatment decision. The global testing in non-squamous histology of EGFR, ALK, and ROS has been 78.9, 64.7, 35.6%, respectively. PDL1 has been globally determined in the same period with 46.9% of testing, although it exceeds 85% when we focus on the last 3 years. Despite current determinations rates being relatively high, especially considering the lack of standard national protocols, these figures are still far from acceptable, which should drive us towards a more efficient organization in the future. In Spain, there is no national standard protocol for central or regional biomarker determination, so it exclusively relies on each hospital and its resources. There is a constant increase in available biomarker-targeted drugs. Therefore, biomarker serial determination should be mandatory due to the high positivity rate of 44.5% (4115) in our patients and the difficulty that entails proper tissue retrieval in lung cancer [5, 6]. Several studies question the cost-effectiveness of NGS compared to a sequential diagnosis [7]; however, most were performed when only three biomarkers were available for testing. At present, NGS would be cost-effective [8] in addition to the shorter time to achieve a complete result, even more so when the determination in blood of these biomarkers by liquid biopsy techniques could reverse the usual tissue limitations [9].

Compared with other countries, Spain accounts for a similar global positivity rate, as the 42% observed in an Italian study [10] and 42.9% in a German study [11], as well as individually the positivity rates by biomarker. Likewise, determining at least one biomarker is similar to the German experience and to the real-life data reported from the USA [12].

The decrease in biomarkers testing observed in the last year is striking, coinciding with the situation experienced by the COVID-19 pandemic. A reduction in both squamous and non-squamous determinations was detected, which suggests that this has been the cause and not a change in the behaviour of the tumour. There has been much speculation about the delay in cancer diagnosis, mostly in early stages, but this probably has affected all stages of the disease, including molecular determinations [13]. This situation is dire given the current availability of targeted treatments that have demonstrated a higher response rate and survival than standard therapy.

As a limitation of the study, it may not exactly reflect the global situation of molecular testing in the country. However, the large cohort of patients studied, from more than 180 hospitals from all over Spain and the continued inclusion of information, may have served to minimize that possible risk. Also, differences between testing rates may be lower in the first years of the period at study as it was not yet a conventional practice in all hospitals and towards 2020 it has become a standard practice.

In our opinion, Spanish hospitals have assumed and performed an adequate level of molecular testing, comparable to other European countries and higher than that in the USA. We believe this demonstrates the strength of our national health system, with universal coverage and the involvement of the physicians, despite the absence of guidelines or governmental organization of these diagnostic aspects. Nevertheless, the complexity of this situation may increase shortly since the presence of new indications linked to biomarkers; the shortage of tumour tissue and the need to obtain a rapid diagnosis in a particularly aggressive disease represent an urgent organizational need at a national level for precision medicine.

## Data Availability

The datasets generated and/or analyzed during the current study are not publicly available due data privacy compliance but are available from the corresponding author on reasonable request.

## References

[1] González M, Calvo V, Redondo I, Provencio M (2021). Overall survival for early and locally advanced non-small-cell lung cancer from one institution: 2000-2017. Clin Transl Oncol.

[2] Gutiérrez L, Royuela A, Carcereny E (2021). Prognostic model of long-term advanced stage (IIIB-IV) EGFR mutated non-small cell lung cancer (NSCLC) survivors using real-life data. BMC Cancer.

[3] Ruano A, Provencio M, Calvo V, et al. Lung cancer symptoms at diagnosis: results of a nationwide registry study. ESMO Open. 2020;5e001021. 10.1136/esmoopen-2020-001021. 10.1136/esmoopen-2020-001021 PMC7678343 33214227

[4] Barquín M, Calvo V, García-García F (2020). Sex is a strong prognostic factor in stage IV non-small-cell lung cancer patients and should be considered in survival rate estimation. Cancer Epidemiol.

[5] Imyanitov EN, Iyevleva AG, Levchenko EV (2021). Molecular testing and targeted therapy for non-small cell lung cancer: current status and perspectives. Crit Rev Oncol Hematol.

[6] Liam CK, Mallawathantri S, Fong K (2020). Is tissue still the issue in detecting molecular alterations in lung cancer?. Respiratory.

[7] Schluckebier L, Caetano R, Garay OU, Montenegro GT, Custodio M, Aran V, Gil FC (2020). Cost-effectiveness analysis comparing companion diagnostic tests for EGFR, ALK, and ROS1 versus next-generation sequencing (NGS) in advanced adenocarcinoma lung cancer patients. BMC Cancer.

[8] Tan AC, Lai GGY, Tan GS (2020). Utility of incorporating next-generation sequencing (NGS) in an Asian non-small cell lung cancer (NSCLC) population: incremental yield of actionable alterations and cost-effectiveness analysis. Lung Cancer.

[9] Romero A, Serna-Blasco R, Calvo V, Provencio M (2021). Use of liquid biopsy in the Care of Patients with non-small cell lung Cancer. Curr Treat Options in Oncol.

[10] Gobbini D, Galetta M, Tiseo M (2017). Molecular profiling in Italian patients with advanced non-small cell lung cancer: an observational prospective study. Lung Cancer.

[11] Griesinger F, Eberhardt W, Nusch A (2021). Biomarker testing in non-small cell lung cancer in routine care: analysis of the first 3717 patients in teh German prospective, observational, nation-wide CRISP registry (AIO-TRK-0315). Lung Cancer.

[12] Robert NJ, Nwokeji ED, Espirito JL, et al. Biomarker tissue journey among patients (pts) with untreated metastatic non-small cell lung cancer (metastatic NSCLC) in the U.S. Oncology Network community practices. J Clin Oncol. 2021;39(suppl 15; abstr 9004). 10.1200/JCO.2021.39.15_suppl.9004.

[13] Provencio M, Mazarico Gallego JM, Calles A (2021). Lung cancer patients with COVID-19 in Spain: GRAVID study. Lung Cancer.

